# 3D-Networks Based Polymer Composites for Multifunctional Thermal Management and Electromagnetic Protection: A Mini Review

**DOI:** 10.3390/ma17102400

**Published:** 2024-05-16

**Authors:** Houbao Liu, Xiaohu Ji, Wei Wang, Lihua Zhou

**Affiliations:** 1 School of Mechanical and Vehicle Engineering, West Anhui University, Lu’an 237012, China; 2 Anhui Province Key Laboratory of Aerospace Structural Parts Forming Technology and Equipment, Hefei University of Technology, Hefei 230009, China; 3 School of Environment and Tourism, West Anhui University, Lu’an 237012, China; qingchunluren@126.com

**Keywords:** electromagnetic interference shielding, thermal conductivity, 3D networks

## Abstract

The rapid development of miniaturized, high-frequency, and highly integrated microelectronic devices has brought about critical issues in electromagnetic compatibility and thermal management. In recent years, there has been significant interest in lightweight polymer-based composites that offer both electromagnetic interference (EMI) shielding and thermal conductivity. One promising approach involves constructing three-dimensional (3D) interconnection networks using functional fillers in the polymer matrix. These networks have been proven effective in enhancing the thermal and electrical conductivity of the composites. This mini-review focuses on the preparation and properties of 3D network-reinforced polymer composites, specifically those incorporating metal, carbon, ceramic, and hybrid networks. By comparing the effects of different filler types and distribution on the composite materials, the advantages of 3D interconnected conductive networks in polymer composites are highlighted. Additionally, this review addresses the challenges faced in the field of multifunctional thermal management and electromagnetic protection materials and provides insights into future development trends and application prospects of 3D structured composites.

## 1. Introduction

Advances in science and technology have greatly contributed to the impressive progress in microelectronic devices, Moore’s Law indicates that modern electronic devices and circuits are developing towards miniaturization and high integration [1,2]. However, this progress has also led to a significant challenge in thermal management due to the accumulation of heat in the local thermal region. This heat accumulation can greatly impact the function, reliability, and lifetime of devices [3,4,5,6,7]. Additionally, the increasing popularity of handheld and wearable micro devices has raised concerns about electromagnetic radiation pollution. Such pollution can have harmful effects on human health and the normal operation of electronic communication equipment [8,9,10,11,12]. Therefore, it is of great significance to develop materials with both high thermal conductivity and electromagnetic interference (EMI) resistance to address these challenges [13,14].

Nowadays, polymer materials are widely used in various industrial fields due to their low cost, excellent chemical stability, and easy processing formability. They have overcome the drawbacks of traditional metal materials such as high cost, easy corrosion, and processing difficulties [15,16,17]. However, most polymer materials are inherently insulating, making them transparent to electromagnetic waves, and their intrinsic thermal conductivity is usually relatively low (0.1 to 0.5 W/m·K). This lack of competitiveness in the field of integrated circuits and microelectronic packaging is due to the limited functions of pure polymers [18,19,20]. To improve the thermal conductivity and EMI shielding performance of polymer materials, researchers have explored the addition of high thermal conductivity and high electrical conductivity fillers to prepare polymer composites [21,22]. These fillers include carbon fibers [23,24], graphene [25,26], carbon nanotubes [27,28], silver nanowires [29,30], etc. However, achieving superior thermal conductivity and EMI shielding performance typically requires a high filler loading [31,32], which inevitably increases manufacturing costs, leads to processing difficulties, and compromises mechanical properties [33]. It remains a great challenge to balance high performance and low filler content costs.

The construction of a three-dimensional (3D) network in the polymer matrix can effectively control the dispersing and distribution of fillers, leading to improved heat and electricity transport. This method has been proven to enhance the performance of polymer composites [34,35]. In conductive materials, thermal conduction is mainly facilitated by free electrons [36], while insulating materials achieve thermal conduction through crystal lattice vibrations known as phonons [37,38]. When a continuous 3D network is formed within a polymer matrix, thermal conductive fillers become interconnected, creating a high-speed channel for the transmission of electrons and phonons. This greatly reduces the thermal resistance at the interface between fillers [39,40], thereby enhancing heat transfer and improving the thermal conductivity of polymer composites. Additionally, the 3D conductive networks effectively connect conductive fillers to facilitate electron transmission [41]. The abundant interfaces also contribute to enhancing the attenuation of electromagnetic waves [42,43], making polymer composites with ultra-low loads of conductive fillers highly effective in EMI shielding. So far, some reviews related to thermal conductive or EMI shielding materials have been reported. Gu et al. [22] analyzed the factors influencing the thermal conductivity of polymers and polymer composites, such as the polymer matrix, fillers, and processing methods. Feng et al. [40] investigated the thermal conductivity performance and mechanism of composites by focusing on the design and preparation of 3D interconnection networks in polymer composites. Aslani et al. [44] reviewed the advancements in novel metal materials and processes for EMI shielding. Gu et al. [45] discussed the design and EMI shielding mechanism of the latest polymer-based EMI shielding materials incorporating 3D conductive networks. However, there is a lack of comprehensive reviews that systematically analyze composites possessing both thermal and EMI shielding properties. Considering the benefits of 3D interconnection networks in improving the thermal conductivity and EMI shielding performance of polymer composites with fillers, it is essential to present the latest progress in this field.

**Figure 1:**
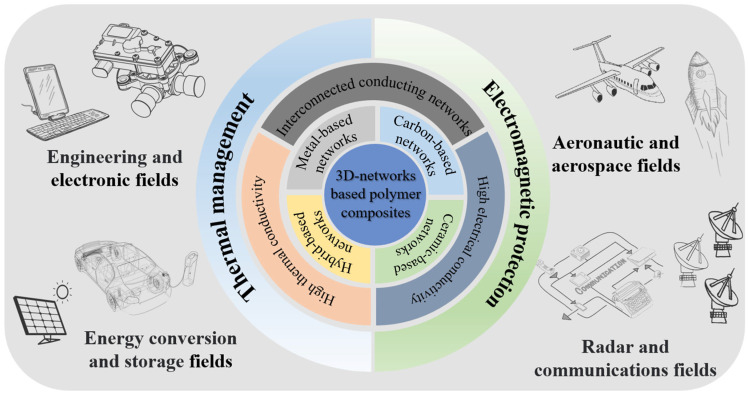
A brief overview of the design strategies and applications of existing 3D-network-based polymer composites.

This mini-review provides an elaboration on the mechanisms of thermal conductivity and EMI shielding in composites. It also summarizes the recent advancements in polymers that exhibit enhanced thermal conductivities and EMI shielding. The main focus is on the enhancement mechanisms of 3D conduction networks formed by different functional fillers such as metals, carbon, ceramics, and hybrid materials, and their impact on the performance of polymer composites. Figure 1 presents a concise overview of the design strategies and applications of existing 3D-network-based polymer composites.

## 2. Thermal Conduction and EMI Shielding Mechanisms of Polymer Composites

### 2.1. Thermal Conduction Mechanism

Polymers are commonly known as thermal insulators due to their predominantly saturated systems with minimal free electrons [46]. Heat conduction in polymers primarily occurs through the thermal vibrations of molecules or atoms in fixed positions, meaning that heat is transferred solely through phonons [47]. However, the presence of disordered polymer molecular chains, chain ends, weak molecular interactions, crystalline–amorphous interfaces, and voids can cause extensive phonon scattering and hinder phonon transmission, resulting in low thermal conductivity [48,49].

**Figure 2:**
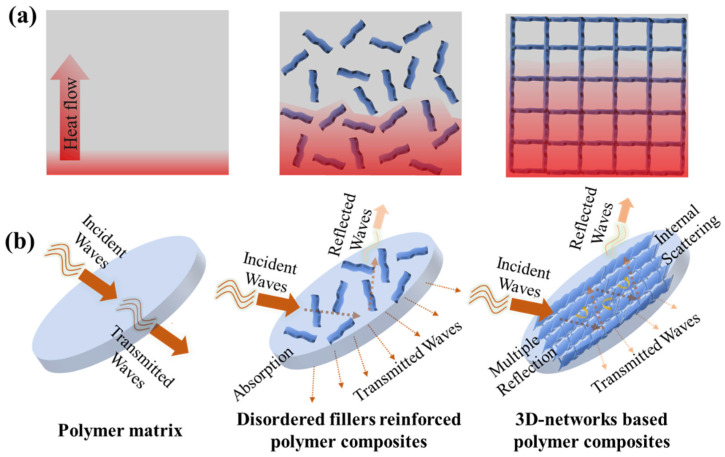
The schematic diagram of (**a**) heat transfer in polymer and polymer composites, (**b**) possible interaction of EM waves with polymer and polymer composites.

Combining polymers with thermally conductive fillers is considered an effective and convenient method for enhancing heat transfer [50]. The mechanism of thermal conduction in polymer-based composites is widely accepted to involve the thermal conduction path and thermal percolation theory [22,51]. The advancement of nanomaterial technology has resulted in the production of various types, forms, sizes, and properties of novel conductive fillers, providing more options for the preparation of thermal conductive polymer composites [52]. The heat flux is transmitted along highly heat-conductive transmission networks formed by thermal conductive fillers in the polymer matrix. The formation of a thermal percolation network of fillers is crucial for improving the thermal conductivity of polymer composites [40,53]. Figure 2a illustrates that compared to pure polymers and randomly dispersed fillers reinforced polymer composites, an ordered 3D network structure is advantageous for manufacturing high-performance polymer composites. This structure ensures filler dispersion and forms an effective interconnected network in the polymer matrix, both of which minimize the interfacial thermal resistance at the filler–filler interface, allowing heat to be transmitted along the low thermal resistance filler network. Moreover, by utilizing this 3D network, significant enhancement in thermal conductivity can be achieved with a low filler loading.

### 2.2. EMI Shielding Mechanism

EMI shielding effectiveness (*SE*) is measured in decibels [dB] and defined as the logarithmic ratio between power (*P*), electric (*E*) and magnetic (*H*) field intensities of the incident to transmitted radiation as [54]:

(1) $$\mathrm{EMI}\;\mathrm{SE}=-10\log\left(\frac{P_{T}}{P_{I}}\right)=-20\log\left(\frac{E_{T}}{E_{I}}\right)=-20\log\left(\frac{H_{T}}{H_{I}}\right)$$

where *P_I_*, *E_I_*, *H_I_* and *P_T_*, *E_T_*, *H_T_* are the incident and transmitted power, electric and magnetic field intensities, respectively. The total *EMI SE* (*SE_T_*) is the sum of attenuation achieved by reflection (*SE_R_*), absorption (*SE_A_*), and multiple reflections (*SE_M_*). The *SE_M_* can be neglected when *SE_T_* is higher than 15 dB. Therefore, *SE_T_ SE* total is shown as Equation (2) [55,56]:

(2) $${\mathrm{SE}}_{T}={\mathrm{SE}}_{R}+{\mathrm{SE}}_{A}$$

Experimentally, the reflection (*R*), absorption (*A*) and transmission (*T*) coefficients were obtained from the network analyzer in the form of scattering parameters (S11, S12, S21, S22), which is used to evaluate how energy is scattered from the shield. They can be expressed as Equations (3)–(5), respectively:

(3) $$R={\left|S11\right|}^{2}={\left|S22\right|}^{2}$$

(4) $$T={\left|S12\right|}^{2}={\left|S21\right|}^{2}$$

(5) A=1−R−T

*SE_R_* and *SE_A_* can be expressed in terms of reflection and effective absorption considering the power of the incident electromagnetic waves inside the shielding material as Equations (6) and (7):

(6) $${\mathrm{SE}}_{R}=-10\log\left(1-R\right)$$

(7) $${\mathrm{SE}}_{A}=-10\log\left(\frac{T}{1-R}\right)$$

Many reported works of literature investigated the shielding mechanism by comparing the contributions of SE_A_ and SE_R_ to the SE_T_ [57,58,59]. If the value of SE_A_ is higher than that of SE_R_, it can be regarded as an absorption mechanism. Since the reflection occurs before the absorption, SE_A_ represents the ability of shieling materials to attenuate electromagnetic waves that only penetrate into the shield [60]. The evaluation of the main shielding mechanism of shielding materials should be based on the value of power coefficients [18]. When A is greater than the R, EMI shielding mainly depends on absorption. Conversely, if R is greater than A, the shielding is mainly due to reflected loss.

Once EM waves propagate from one medium to another, the most noticeable interaction is reflection, which occurs due to impedance mismatch. Conductive materials with movable charge carriers can effectively reflect EM waves [61]. The remaining EM waves enter the interior of the material, where they interact with the electrical and/or magnetic components of the dipoles present, converting electromagnetic energy into heat absorption [62]. Another possible interaction of EM waves within the material is multiple reflections, particularly in porous materials and 3D-network composites, leading to the scattering or multiple reflections of EM waves [63]. Transmitted waves that are neither reflected nor absorbed can penetrate the material. Figure 2b illustrates the potential interaction of EM waves with polymers and polymer composites.

## 3. 3D-Network-Based Polymer Composites with High Thermal Conductivity and EMI Resistance

The performance of 3D-network-based polymer composites, which aim to enhance both thermal conductivity and EMI shielding, primarily depends on the inner filler networks. These networks can be classified into four categories based on the type of fillers used: metal networks, carbon material networks, ceramic networks, and hybrid networks.

### 3.1. 3D Metal Network-Based Polymer Composites

**Figure 3:**
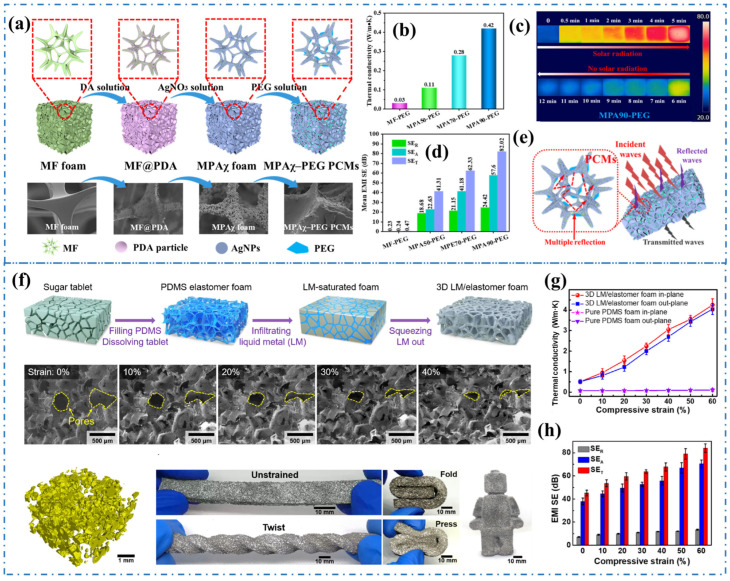
(**a**) Schematic diagram of the preparation of MPAχ-PEG PCMs, (**b**) The thermal conductivity of MF-PEG and MPAχ-PEG PCMs, (**c**) Infrared thermography images of MPAχ-PEG PCMs under solar irradiation, (**d**) The EMI SE of MPAχ-PEG PCMs, (**e**) Illustration of EM waves transmission and attenuation in the MPAχ-PEG PCMs [67]. Copyright 2022, Elsevier. (**f**) Schematic illustration of the fabrication method of the 3D LM/elastomer foam, (**g**) The thermal conductivity of 3D LM/elastomer foam, (**h**) The EMI SE of 3D LM/elastomer foam [68]. Copyright 2021, Elsevier.

Metals have been extensively utilized as heat transfer and EMI shielding materials owing to their exceptional thermal and electrical conductivity. Nevertheless, the majority of metals possess drawbacks such as being heavy, expensive, corrosive, and challenging to process. Constructing an interconnected network in a polymer matrix with metal fillers can effectively address the limitations of pure metals in various applications. This approach leads to significant enhancements in thermal conductivity and EMI shielding performance [64,65]. Lee et al. [66] utilized electroless plating Cu/Ag on PS beads and hot pressing technology to prepare 3D metal-coated polystyrene (PS) shell network composites. The obtained interconnected Cu and Ag shell network reduces contact resistance and interface resistance, thereby improving electrical and thermal conductivity. The metal shell networks facilitate efficient electron and heat conduction within the composites, resulting in an impressive EMI SE of 110 dB at a thickness of 0.5 mm and a high thermal conductivity of 16.1 W/(m·K) with 13 vol % of metal filler loading. Xiao et al. [67] employed a mussel-inspired strategy to construct a flexible melamine foam (MF) wrapped with polydopamine (PDA) and silver nanoparticles (AgNPs). Additionally, they prepared MPAχ-PEG phase change materials (PCMs) by wrapping polyethylene glycol (PEG). The continuous AgNPs wrapped on the MF skeleton form an interconnection network for heat and electricity transmission. Compared to MF-PEG, the MPAχ-PEG PCMs exhibited a relatively high phase transition enthalpy of 148.9 J/g and a remarkable thermal conductivity enhancement of 1400% (Figure 3b). Furthermore, the metallized MPAχ foams endowed MPAχ-PEG PCMs with a high electrical conductivity of 223.7 S/m, enabling excellent electrical to thermal energy conversion and storage (Figure 3c) with a conversion efficiency of approximately 86.3%. Due to the multiple reflection attenuation of EM waves in the porous metal network, MPAχ-PEG PCMs also demonstrate efficient EMI SE, with a SE_T_ of 82.02 dB in the X-band (Figure 3d,e).

The preparation of flexible and deformable metal materials that can effectively shield both EMI and dissipate heat poses a significant challenge in practical applications. Yao et al. [68] developed flexible composites by introducing a 3D liquid metal (LM, Ga and In) network into the elastomer foam. This resulted in composites with significantly increased electrical/thermal conductivities and EMI SE under compression (Figure 3f). The 3D LM/elastomer foam maintained stable performance in 10,000 compression release cycles at 50% strain, with no observed microstructure damage or performance degradation, demonstrating its exceptional compliance, robustness, and stability. The thermal conductivity and EMI shielding properties of the 3D LM/elastomer composites showed a positive correlation with the compressive strain. At 60% strain, the in-plane thermal conductivity reached 4.25 W/(m·K) and the EMI SE reached 85 dB (Figure 3g,h). By fully utilizing the superior thermal and electrical conductivity of metal materials in polymer-based composites, the 3D metal network overcomes the drawbacks of heavy weight and easy corrosion, making it an ideal filler for thermal management and EMI shielding composites.

### 3.2. 3D Carbon Network-Based Polymer Composites

**Figure 4:**
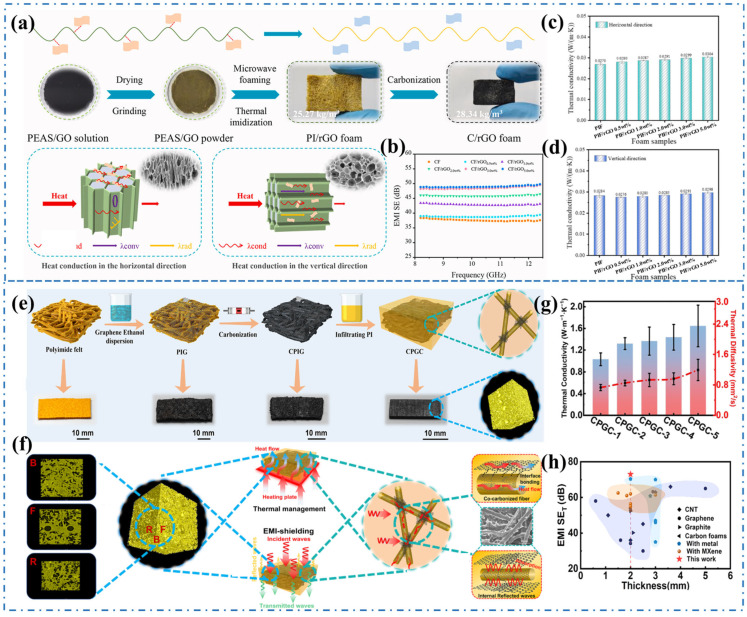
(**a**) Schematic diagram of the preparation and thermal insulation mechanism of the composite foams, (**b**) The EMI SE of the composite foams, (**c**,**d**) The thermal conductivity of the composite foams in the vertical and horizontal directions [75]. Copyright 2023, Elsevier. (**e**) Schematic illustration of the fabrication of 3D graphene/carbon fiber reinforced polyimide composites, (**f**) Illustration of EMI shielding and thermal management in the polyimide composites, (**g**) The thermal conductivity of the polyimide composites, (**h**) The EMI SE of the polyimide composites [77]. Copyright 2023, Elsevier.

The fascinating characteristics of carbon-based nanomaterials, such as their lightweight nature, corrosion resistance, and excellent electrical and thermal properties, make them highly promising for various applications [69,70]. Carbon nanotubes (CNTs) and graphene, along with carbon nanofibers (CNFs) and nanoscale carbon black (CB), are widely utilized as conductive fillers in EMI shielding and thermal management composites [71,72,73]. Kumar et al. [74] conducted a study that impregnated phenolic resin and cenospheres in a polyurethane (PU) foam matrix, and then heat treated at 1000 °C to convert the impregnated foam into a carbon microsphere composite foam using the sacrificial template method. The resulting carbon-cenosphere composite foams had a low density ranging from 0.30 to 0.45 g/cm^3^ and an open cell structure. The EMI SE of the composite foams increased with an increase in cenosphere content. In the frequency range of 8.2–12.4 GHz, the foam loaded with 30.0 wt.% cenospheres exhibited the highest absorption of −42.9 dB. Additionally, the composite foams demonstrated excellent thermal insulation properties, with a low thermal conductivity of 0.02 W/(m·K). Ni et al. [75] proposed a facile method using microwave assistance for foaming and thermal imidization to prepare flexible 3D polyimide (PI)/rGO composite foams with an anisotropic pore structure. The directional growth of the 3D porous structure enhances the mechanical properties, thermal insulation, and EMI SE of the composite foams, as depicted in Figure 4. The electrical conductivity and EMI SE of the foams increase with the rGO content. In the horizontal direction, the foam achieved an electrical conductivity of 33.0 S/m, EMI SE of 49.0 dB, and specific EMI SE of 8792.8 dB/(g/cm^2^). The thermal conductivity of foams initially decreases and then increases in the vertical direction, because the low content of rGO increases the interface thermal resistance and phonon scattering, leading to a decrease in thermal conductivity. At the same time, it shows an increasing trend in the horizontal direction due to rGO forming a better “heat transfer path” through the interconnection structure of the PI skeleton. Fan et al. [76] fabricated ultra-thin graphene nanosheet-based foams with micropores using the traditional salt template-guided freeze-drying-calcination method. In the C, S, and Ku bands, the EMI SE exceeds 20 dB with a low filler loading of 7 wt%. Additionally, the 3D interconnect framework reduces the contact thermal resistance between nanosheets and provides a continuous pathway for thermal and electrical conduction. The foams exhibit higher thermal conductivity (3.26–3.95 W/(m·K)) at lower filling ratios (3–5 wt%).

The structural diversification of carbon materials leads to a wide range of properties, and the combination of carbon fillers with different structures can often endow composites with better properties. Li et al. [77] introduced a co-carbonization strategy to create a 3D graphene/carbon fiber interconnect network, enhancing the EMI shielding performance and thermal conductivity of polyimide composites (Figure 4e). The composites prepared with a 20 wt% content of the 3D carbon network exhibited an impressive EMI SE of 73 dB at a thickness of 2 mm (Figure 4h). In addition, the through-plane thermal conductivity of the composites reached 1.65 W/(m·K), which is 611% higher than that of the polymer matrix (Figure 4g). The co-carbonization strategy of graphene and polyimide carbonized fibers created a robust electron and phonon transport network within the polyimide matrix, establishing π-π conjugation between the interfaces, which is beneficial for improving the EMI shielding and thermal conductivity of the composites, as shown in Figure 4f. Similarly, polydimethylsiloxane (PDMS) composites reinforced with a dual 3D structure of graphene nanosheets and fluorinated graphene exhibited excellent EMI shielding performance of 51.26 dB and high thermal conductivity of 1.47 W/(m·K) [78]. These structure–function integration strategies for constructing 3D carbon networks contribute to the development of novel high-performance bifunctional composites for aerospace and military applications.

### 3.3. 3D Ceramic Network-Based Polymer Composites

**Figure 5:**
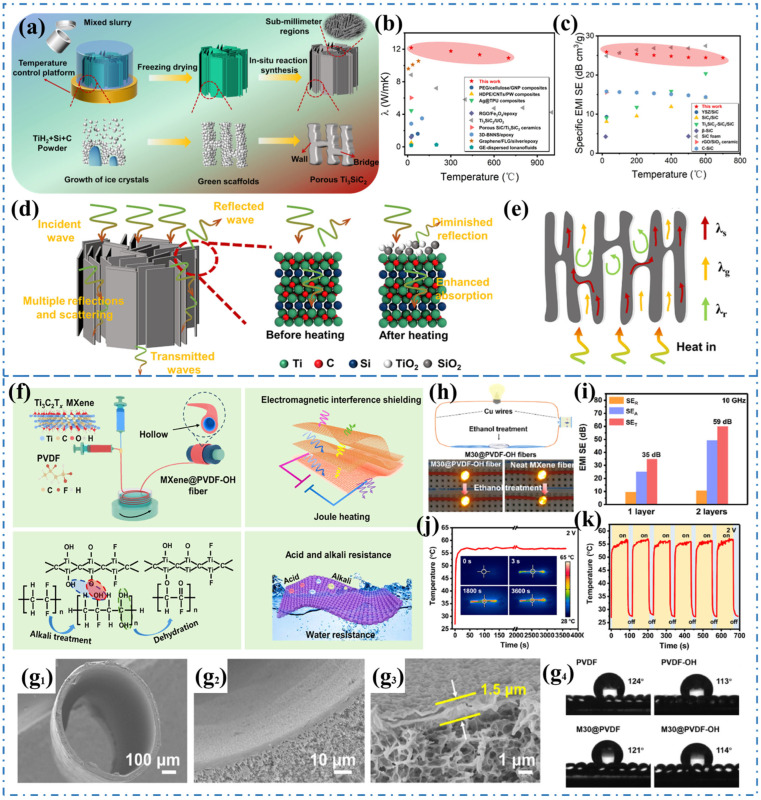
(**a**) Schematic diagram of the preparation of porous Ti_3_SiC_2_ ceramics, (**b**,**c**) The EMI SE and thermal conductivity of porous Ti_3_SiC_2_ ceramics, (**d**,**e**) Illustration of EMI shielding and heat transfer 3D porous ceramic [84]. Copyright 2023, Elsevier. (**f**) Schematic illustration of the fabrication of 3D hollow core–shell MXene/PVDF fibers, (**g_1_**–**g_4_**) Cross-sectional SEM images and contact angles of MXene/PVDF fibers, (**h**,**i**) The electrical conductivity and EMI shielding performance of MXene/PVDF fibers, (**j**,**k**) The Joule heating performance of MXene/PVDF fibers [94]. Copyright 2023, Elsevier.

Although various composites such as metal-based, carbon-based, and polymer-based materials are commonly used for EMI shielding and thermal management, their limited resistance to oxidation and susceptibility to failure restrict their application in high-temperature and high-humidity environments [79,80,81]. However, in aerospace and offshore oil field applications, there is a need for electronic components that can withstand long-term use in high-temperature and corrosive environments. This is where ceramic materials with exceptional properties such as super high-temperature resistance, corrosion resistance, and thermal conductivity have a natural advantage in providing electromagnetic protection and thermal management in extreme conditions [82,83]. Yang et al. [84] conducted a study in which they synthesized lightweight porous Ti_3_SiC_2_ ceramics using aqueous dispersions of TiH_2_, Si, and C powders through a series of processes including unidirectional freezing, freeze-drying, and in-situ reaction, as depicted in Figure 5a. The ice template method results in a 3D-ordered laminar arrangement of porous Ti_3_SiC_2_ ceramics. When EM waves interact with these porous ceramics, the mismatch in impedance between the ceramic surface and air causes the reflection of the waves. Partially incident waves are converted and absorbed through polarization loss and conductivity loss. Furthermore, the 3D porous structure of the ceramics allows for multiple scattering and reflection of electromagnetic radiation, increasing the chances of wave absorption and resulting in an efficient EMI shielding capability of 35.44 dB for porous Ti_3_SiC_2_ ceramics (Figure 5c,d). Additionally, the continuous ceramic array structure formed through unidirectional freezing shortens the heat transfer path, leading to a high thermal conductivity of 12.17 W/(m·K) (Figure 5b,e). Similar research in the near-net-formed Ti_3_SiC_2_/SiC composites was prepared by a combination of molten salt synthesis and polymer infiltration pyrolysis [85]. The continuous and uniform distribution of the 3D Ti_3_SiC_2_ skeleton can effectively improve the mechanical properties, EMI SE, and thermal conductivity of Ti_3_SiC_2_/SiC composites. Compared with polymer-derived SiC bulk ceramic, the bending strength and fracture toughness of composites increased by 53.3% and 95.7%, respectively. The EMI SE exhibited an average value of 46.92 dB across the entire X-band, while the thermal conductivity ranged from 6–7 W/(m·K) over a temperature range of 25–1000 °C.

2D MXene is a special type of transition metal carbide/nitride/carbonitride, typically prepared by selectively extracting certain atoms from their layered ceramic matrix (such as MAX phase) [86,87]. MXene offers several advantages, including good conductivity, a large specific surface area, and lightweight properties, making it highly promising for applications in EMI shielding and thermal management [88,89,90,91]. In a study conducted by Alshareef et al. [92], MXene membranes were prepared using vacuum-assisted filtration, resulting in high electrical conductivity and moderate n-type Seebeck coefficients. Mo_2_TiC_2_T_x_, for instance, exhibited a thermoelectric power of 3.09 × 10^−4^ W/(m·K^2^) at 803 K. Another research by Han et al. [93] involved the creation of a tunable 3D porous MXene aerogel with exceptional EMI shielding performance. This was achieved through a bidirectional freezing and freeze-drying process. The study revealed that the shielding mechanism of Ti_3_C_2_T_x_ MXene primarily relies on dielectric loss. Ye et al. [94] utilized a coaxial wet-spinning assembly strategy to prepare MXene-coated polyvinylidene fluoride (PVDF) 3D hollow core–shell fibers, which demonstrated remarkable hydrophobic properties. The interaction between the MXene core and PVDF shell was effectively enhanced by utilizing partially hydroxylated PVDF obtained from alkaline treatment, as depicted in Figure 5f,g_1_–g_4_. The ordered MXene layers forming the hollow core contributed to a high electrical conductivity of 3.08 × 10^5^ S/m for the fibers. Moreover, the 3D hollow core–shell structure facilitated multiple scattering of EM waves, leading to increased absorption and attenuation, resulting in superior EMI SE of 60 dB (Figure 5h,i). Furthermore, the MXene-enhanced PVDF 3D hollow fiber exhibited excellent performance in low voltage-driven Joule heating for human thermal management (Figure 5j,k). The development of high-performance composite materials reinforced with specialized ceramic fillers is expected to play a crucial role in controlling electromagnetic radiation pollution, thermal management, and energy storage.

### 3.4. 3D Hybrid Network-Based Polymer Composites

**Figure 6:**
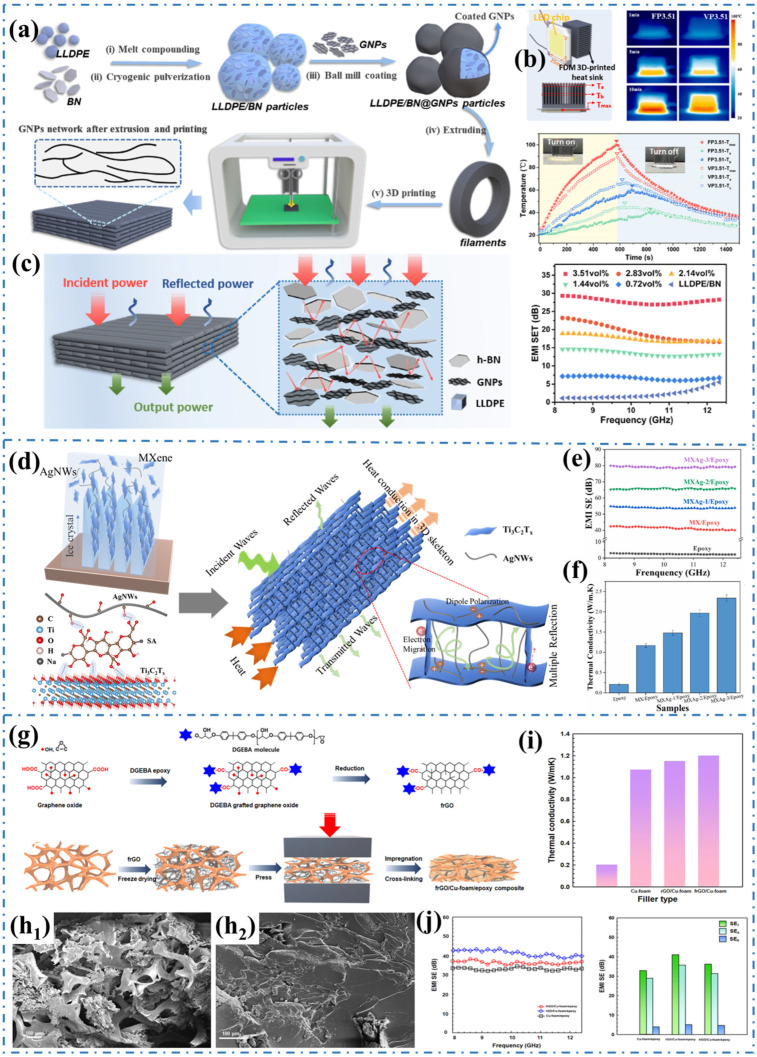
(**a**) Schematic diagram of the preparation of LLDPE/BN@GNPs composites, (**b**) The display of heat dissipation performance of composites, (**c**) The EMI shielding performance of composites [105]. Copyright 2023, Elsevier. (**d**) Schematic diagram of MXene/AgNWs/Epoxy composites and illustration of EMI shielding and heat transfer, (**e**,**f**) The EMI shielding and thermal conductivity performance of MXene/AgNWs/Epoxy composites [107]. Copyright 2022, Elsevier. (**g**) Schematics of the preparation of graphene–copper/polymer composites, (**h_1_**,**h_2_**) The SEM images of graphene–copper/polymer composites, (**i**) The thermal conductivity and (**j**) The EMI shielding performance of graphene–copper/polymer composites [110]. Copyright 2022, Elsevier.

A significant amount of research has shown that incorporating multiple fillers into polymers is an effective strategy for achieving high-performance composites [40,95,96,97,98,99,100]. By combining various filling materials with different scales, shapes, and properties through a well-designed process, significant synergistic effects can be achieved compared to 3D networks constructed with single fillers [101,102]. These 3D hybrid networks greatly improve the thermal and electrical conductivity of polymer-based composites [103,104]. For instance, Peng et al. [105] designed a conductive dual network system using graphene nanosheets (GNPs) and boron nitride (BN) conductive networks. They employed ball milling strategy and fused deposition modeling (FDM) 3D printing technology to achieve high thermal conductivity and EMI shielding in low-density polyethylene (LLDPE) composites, as shown in Figure 6a. The GNP network acts as a bridge connecting the BN filler network, thereby increasing the density of the heat conduction network. The heat sink used for thermal management can be printed with different filler arrangements to maximize its thermal conductivity utilization. The thermal conductivity of the printed VP3.51 and FP3.51 components is as high as 3.11 W/(m·K) and 1.02 W/(m·K), respectively (Figure 6b). Additionally, the formed GNP network promotes rapid carrier migration, resulting in LLDPE/BN@GNPs composites with good EMI shielding performance of 27.8 dB (Figure 6c). More importantly, anisotropic or isotropic conductive composites can be designed by controlling the 3D printing conditions (flat or vertical printing). A Ti_3_C_2_T_x_/GO framework with vertical pore gradient was constructed using 3D printing technology, and a TiO_2_-Ti_3_C_2_T_x_ heterojunction was generated in situ through thermal annealing. By curing the 3D framework with polydimethylsiloxane (PDMS), TiO_2_-Ti_3_C_2_T_x_/rGO/PDMS composites with high EMI SE and excellent thermal management performance can be assembled [106].

The ideal transmission of phonon electrons is facilitated by the continuous network created through the combination design of multi-scale functional fillers. In a study by Liu et al. [107], a 3D MXene/AgNWs aerogel with an interpenetrating double network structure was developed using directional freezing and freeze-drying technology. To address the weak gelling of nano fillers, biocompatible sodium alginate, rich in oxygen-containing functional groups, forms strong hydrogen bonds between 1D AgNWs and 2D MXene. Subsequently, MXene/AgNWs/epoxy nanocomposites were produced by vacuum impregnation and curing, utilizing the hybrid aerogel as a reinforced skeleton (Figure 6d). The interconnected hierarchical microstructure constructed by MXene and AgNWs minimizes contact thermal resistance between fillers, creating more efficient channels for electron and phonon transport. Furthermore, the continuous conductive network with a porous structure enables effective attenuation of EM waves through multiple reflections and absorption. The resulting MXene/AgNWs/Epoxy nanocomposites exhibit an EMI SE value of 94.1 dB and a thermal conductivity of 2.34 W/(m·K) with a low MXene/AgNWs content of 8.2 wt% (Figure 6e,f). Gu et al. [108] used a two-step vacuum filtration and hot pressing method to combine spherical MOF-derived CoNi@C with 1D silver nanowires and cellulose nanofiber, and designed composites with outstanding EMI shielding and thermal management performance. Huang et al. [109] modified the surface of 2D MXene nanosheets with 0D silver nanoparticles through in-situ reduction and prepared composite films with micro 3D structures using naturally abundant nanocellulose fibers as the matrix. The prepared composite films exhibit a high thermal conductivity of 7.31 W/(m·K) and an EMI SE value of 25.8 dB.

The multiple interconnected networks constructed by combining hybrid fillers can provide multiple percolation channels for electricity and heat. Lee et al. [110] grafted diglycidyl ether of bisphenol-A onto graphene surfaces, resulting in the formation of graphene–copper double 3D networks through freeze-drying. The double 3D networks were pressed and wetted with epoxy resin to form polymer-based composites (Figure 6g). Cu-foam and graphene were used as thermal and electric conductive fillers of the polymer matrix. Primary Cu-foam acted as the basic skeleton of the 3D structure, while secondary filler graphene served as the bridge between the Cu-foam, creating the double networks, as shown in Figure 6h_1_,h_2_. The composites reinforced by these dual networks exhibited a thermal conductivity of 1.2 W/(m·K) and an EMI SE of 38.16 dB, surpassing the reinforcement effect of a single Cu network (Figure 6i,j). Gao et al. [111] also utilized carbonized melamine foam and graphene to construct a double 3D conductive synergistic network. They prepared carbon foam/reduced graphene oxide/paraffin composite phase change materials through reduction assembly and vacuum impregnation. Due to the enhancement of the dual 3D interconnection networks, the EMI SE of the composites reaches 49 dB, and the thermal conductivity is 324% higher than pure paraffin. These hybrid 3D networks enhanced polymer-based composites possess enormous application potential in the fields of thermal management and electromagnetic protection.

## 4. Summary and Perspectives

High-performance polymer-based composites with dual functionalities of thermal management and EMI shielding play a crucial role in the development of the next generation of high-frequency, high-power, and highly integrated electronic devices. The revolutionary advances in nanomaterials and processing technologies have led to the emergence of various innovative fillers and 3D structures, offering numerous possibilities for the fabrication of high-performance polymer-based composites. In this review, we comprehensively discuss the preparation process, performance, and enhancement mechanisms of polymer composites reinforced with metal, carbon, ceramic, and hybrid 3D networks, focusing on their thermal conduction and EMI shielding mechanisms. By analyzing the published literature, Table 1 summarizes the different types of fillers, filler distribution, electrical conductivity, EMI SE, and thermal conductivity of polymer-based composites with varying structures. Compared to randomly distributed fillers and interlayer-ordered structures, 3D-network-based polymer composites can often provide greater EMI SE and thermal conductivity under low filler loading.

Although the construction of 3D networks of fillers can significantly improve the performance of polymer-based composites, there are also some technical issues and challenges. The 3D network structure typically enhances EMI shielding and thermal management capabilities, but at the expense of thickness. Therefore, there is a trade-off between effective performance improvement and thickness. Additionally, the complex preparation and processing methods of 3D networks lead to increased costs and may result in imbalanced mechanical and thermoelectric properties of the composites. In light of these considerations, we propose exploring and developing economical and environmentally friendly methods for constructing 3D networks in the future. Furthermore, the integrated design of composite structure and function, as well as the integration and application demonstration of 3D enhanced thermal conductivity and EMI shielding polymer-based composites in devices, should be further investigated. With advancements in basic theories and advanced processing technologies, it is anticipated that functional composites with 3D conductive networks for thermal management and EMI shielding will play a significant role in fields such as electronic communication, aerospace, energy storage, and environmental protection in the near future.

## Figures and Tables

**Table 1:** Comprehensive analysis of various polymeric composites and their EMI SE, electrical conductivity and thermal conductivity.

Matrix	Filler	Filler Distribution	σ_AC_ (S/m)	EMI SE (dB)	TC (W/m·K)	Ref.
PVDF	50 wt% Ti_3_C_2_T_x_	Random	0.988	34.49	0.767	[13]
Epoxy	15 wt% CNT/Fe_3_O_4_/Ag	Random	28	35	0.46	[112]
Epoxy	8.97 wt% rGO/Fe_3_O_4_	Random	-	13.45	1.213	[113]
Epoxy/PEG	30 wt% MXene	Random	1.3	64.7	0.74	[114]
Silicone Rubber	23.3 vol% Ag@s-BN	Random	3.5 × 10^−12^	20.9	1.53	[115]
POM	40 wt% MWCNT	Random	3484	45.7	1.95	[116]
POM	48 wt% GNP	Random	2695	44.7	4.24	[116]
Polyacrylate	20 wt% Graphene	Random	442.5	58	1.68	[117]
Epoxy	25 vol% LMPA/BNNS	Layered	1.01 × 10^−9^	14	1.06	[118]
PU	60 wt% GNP	Layered	2546.5	67.6	41.6	[119]
Heterocyclic Aramid	80 wt% MXene	Layered	13,811.4	43.0	11.5	[120]
PDMS	10 wt% Ti_3_C_2_T_x_/20 wt% BN	Layered	3.45 × 10^−15^	35.2	0.65	[121]
PEG	13.78 wt% MXene/Graphene	3D	1996	56.6	11.39	[122]
PVDF	10 wt% MWCNTs/rGO/FeCo	3D	6 × 10^−2^	41.2	0.75	[123]
Epoxy Acrylic	15 wt% SCF 6 wt% GNP	3D	0.53	45.93	2.13	[124]
PS	13 vol % Cu/Ag	3D	3.5 × 10^−4^	110	16.1	[66]
PI	20 wt% Graphene/Carbon fiber	3D	3331	73	1.65	[77]
Epoxy	20 wt% AgNS	3D	89.12	42.5	0.268	[125]
Epoxy	8.2 wt% Ti_3_C_2_T_x_/AgNWs	3D	1532	79.3	2.34	[107]
Epoxy	3.55 wt% rGO/CNT/VG	3D	81.88	56.65	2.46	[126]
Silicone Rubber	2.77 wt% MWCNTs /Graphene	3D	7.65	42.0	1.30	[127]
LLDPE	24.89 vol% Graphite	3D	4000	52.4	19.6	[128]

## Data Availability

Not applicable.
